# *trans*-Diaqua­bis­(cyclo­hexane-1,2-diamine)­zinc(II) dichloride

**DOI:** 10.1107/S1600536811008166

**Published:** 2011-03-12

**Authors:** Kamelia Karimnejad, Hamid Khaledi, Hapipah Mohd Ali

**Affiliations:** a Department of Chemistry, University of Malaya, 50603 Kuala Lumpur, Malaysia

## Abstract

In the title compound, [Zn(C_6_H_14_N_2_)_2_(H_2_O)_2_]Cl_2_, the Zn(II) atom resides on a special position with site symmetry 2/*m* and is octa­hedrally coordinated by four N atoms from two *trans* 1,2-diamino­cyclo­hexane ligands and two water O atoms. In the crystal, N—H⋯Cl and O—H⋯Cl hydrogen bonds link the mol­ecules into a two-dimensional network parallel to the *bc* plane.

## Related literature

For an isotypic nickel(II) complex, see: Capilla *et al.* (1980 ▶) and for an analogous copper(II) complex, see: Pariya *et al.* (1998 ▶).
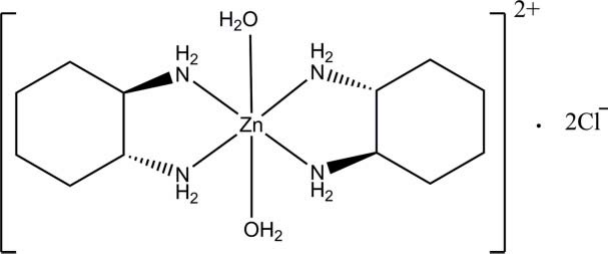


## Experimental

### 

#### Crystal data

[Zn(C_6_H_14_N_2_)_2_(H_2_O)_2_]Cl_2_
*M* *_r_* = 400.69
Orthorhombic, 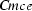

*a* = 24.6478 (4) Å
*b* = 9.5107 (2) Å
*c* = 7.6723 (2) Å
*V* = 1798.52 (7) Å^3^
*Z* = 4
Mo *K*α radiation
μ = 1.67 mm^−1^
*T* = 100 K
0.26 × 0.21 × 0.04 mm

#### Data collection

Bruker APEXII CCD diffractometer
Absorption correction: multi-scan (*SADABS*; Sheldrick, 1996 ▶) *T*_min_ = 0.670, *T*_max_ = 0.936
4324 measured reflections
999 independent reflections
805 reflections with *I* > 2σ(*I*)
*R*_int_ = 0.019

#### Refinement

*R*[*F* ^2^ > 2σ(*F* ^2^)] = 0.021
*wR*(*F* ^2^) = 0.053
*S* = 1.04
999 reflections
61 parameters
3 restraints
H atoms treated by a mixture of independent and constrained refinement
Δρ_max_ = 0.36 e Å^−3^
Δρ_min_ = −0.24 e Å^−3^

### 

Data collection: *APEX2* (Bruker, 2007 ▶); cell refinement: *SAINT* (Bruker, 2007 ▶); data reduction: *SAINT*; program(s) used to solve structure: *SHELXS97* (Sheldrick, 2008 ▶); program(s) used to refine structure: *SHELXL97* (Sheldrick, 2008 ▶); molecular graphics: *X-SEED* (Barbour, 2001 ▶); software used to prepare material for publication: *SHELXL97* and *publCIF* (Westrip, 2010 ▶).

## Supplementary Material

Crystal structure: contains datablocks I, global. DOI: 10.1107/S1600536811008166/pv2395sup1.cif

Structure factors: contains datablocks I. DOI: 10.1107/S1600536811008166/pv2395Isup2.hkl

Additional supplementary materials:  crystallographic information; 3D view; checkCIF report

## Figures and Tables

**Table 1:** Hydrogen-bond geometry (Å, °)

*D*—H⋯*A*	*D*—H	H⋯*A*	*D*⋯*A*	*D*—H⋯*A*
N1—H1*A*⋯Cl1^i^	0.87 (1)	2.80 (1)	3.6139 (13)	156 (1)
N1—H1*B*⋯Cl1	0.87 (1)	2.70 (1)	3.5206 (14)	157 (1)
O1—H1*O*⋯Cl1	0.83 (1)	2.26 (1)	3.0857 (12)	173 (2)

Symmetry code: (i) 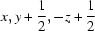.

**Fig. 1.**
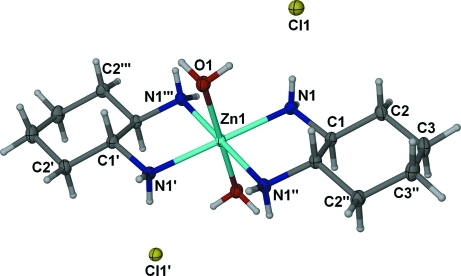
Thermal ellipsoid plot of the title compound at the 50% probability level. Hydrogen atoms are drawn as spheres of arbitrary radius. Symmetry codes: ' = -x, -y, -z; " = x, -y, -z; "' = -x, y, z.

## supplementary crystallographic information

### Comment

The title Zn^II^ complex (Fig. 1) is isostructural with a previously reported Ni^II^ complex (Capilla *et al.*, 1980). The metal center in the title complex is located on a special position with site symmetry 2/m, and is six-coordinated in a distorted octahedral geometry. The equatorial plane is defined by four N atoms from two *trans*-1,2-diaminocyclohexane, while the axial positions are occupied by two water molecule oxygen atoms. This arrangement is similar to what was observed in an analogous Cu^II^ complex (Pariya *et al.*, 1998). In the crystal, an O—H···Cl interaction connects the cationic complexes and chloride anions into infinite chains along the *c*-axis. The chains are further linked by N—H···Cl hydrogen bonds into layers parallel to the *bc* plane.

### Experimental

The colorless crystals of the title compound were obtained upon slow evaporation of an ethanolic solution (20 ml) of *trans*-l,2-diaminocyclohexane (0.23 g, 2 mmol) and zinc(II) acetate dihydrate (0.22 g, 1 mmol) in the presence of a few drops of HCl (37%).

### Refinement

The C-bound hydrogen atoms were placed at calculated positions with C—H = 0.99 and 1.00 Å for methylene and methyne type H-atoms, respectively. The hydrogen atoms bonded to N and water molecule were located from a difference map and included with restraints: O—H = 0.84 (2) and N—H = 0.88 (2) Å. For hydrogen atoms U*iso*(H) were set to 1.2–1.5 times U*eq*(carrier atom).

### Crystal data

**Table:**

[Zn(C_6_H_14_N_2_)_2_(H_2_O)_2_]Cl_2_	*F*(000) = 848
*M**_r_* = 400.69	*D*_x_ = 1.480 Mg m^−^^3^
Orthorhombic, *C**m**c**e*	Mo *K*α radiation, λ = 0.71073 Å
Hall symbol: -C 2bc 2	Cell parameters from 2315 reflections
*a* = 24.6478 (4) Å	θ = 3.3–30.5°
*b* = 9.5107 (2) Å	µ = 1.67 mm^−^^1^
*c* = 7.6723 (2) Å	*T* = 100 K
*V* = 1798.52 (7) Å^3^	Prism, colorless
*Z* = 4	0.26 × 0.21 × 0.04 mm

### Data collection

**Table:**

Bruker APEXII CCD diffractometer	999 independent reflections
Radiation source: fine-focus sealed tube	805 reflections with *I* > 2σ(*I*)
graphite	*R*_int_ = 0.019
φ and ω scans	θ_max_ = 27.0°, θ_min_ = 3.3°
Absorption correction: multi-scan (*SADABS*; Sheldrick, 1996)	*h* = −31→31
*T*_min_ = 0.670, *T*_max_ = 0.936	*k* = −11→12
4324 measured reflections	*l* = −9→8

### Refinement

**Table:**

Refinement on *F*^2^	Primary atom site location: structure-invariant direct methods
Least-squares matrix: full	Secondary atom site location: difference Fourier map
*R*[*F*^2^ > 2σ(*F*^2^)] = 0.021	Hydrogen site location: inferred from neighbouring sites
*wR*(*F*^2^) = 0.053	H atoms treated by a mixture of independent and constrained refinement
*S* = 1.04	*w* = 1/[σ^2^(*F*_o_^2^) + (0.0239*P*)^2^ + 1.6417*P*] where *P* = (*F*_o_^2^ + 2*F*_c_^2^)/3
999 reflections	(Δ/σ)_max_ < 0.001
61 parameters	Δρ_max_ = 0.36 e Å^−^^3^
3 restraints	Δρ_min_ = −0.23 e Å^−^^3^

### Special details

**Table:**

Geometry. All e.s.d.'s (except the e.s.d. in the dihedral angle between two l.s. planes) are estimated using the full covariance matrix. The cell e.s.d.'s are taken into account individually in the estimation of e.s.d.'s in distances, angles and torsion angles; correlations between e.s.d.'s in cell parameters are only used when they are defined by crystal symmetry. An approximate (isotropic) treatment of cell e.s.d.'s is used for estimating e.s.d.'s involving l.s. planes.
Refinement. Refinement of *F*^2^ against ALL reflections. The weighted *R*-factor *wR* and goodness of fit *S* are based on *F*^2^, conventional *R*-factors *R* are based on *F*, with *F* set to zero for negative *F*^2^. The threshold expression of *F*^2^ > σ(*F*^2^) is used only for calculating *R*-factors(gt) *etc*. and is not relevant to the choice of reflections for refinement. *R*-factors based on *F*^2^ are statistically about twice as large as those based on *F*, and *R*- factors based on ALL data will be even larger.

### Fractional atomic coordinates and isotropic or equivalent isotropic displacement parameters (Å^2^)

**Table:**

	*x*	*y*	*z*	*U*_iso_*/*U*_eq_	
Zn1	0.0000	0.0000	0.0000	0.01354 (11)	
O1	0.0000	−0.10232 (16)	0.2580 (2)	0.0194 (3)	
H1O	0.0266 (6)	−0.0795 (18)	0.318 (2)	0.029*	
N1	0.06632 (5)	0.13128 (13)	0.07888 (18)	0.0163 (3)	
H1A	0.0617 (6)	0.2190 (15)	0.050 (2)	0.020*	
H1B	0.0698 (7)	0.1249 (17)	0.1918 (18)	0.020*	
C1	0.11721 (5)	0.08041 (15)	−0.0016 (2)	0.0168 (3)	
H1	0.1174	0.1111	−0.1263	0.020*	
C2	0.16808 (5)	0.13853 (17)	0.0850 (2)	0.0205 (3)	
H2A	0.1678	0.1136	0.2102	0.025*	
H2B	0.1681	0.2423	0.0757	0.025*	
C3	0.21934 (6)	0.08004 (18)	0.0001 (2)	0.0255 (4)	
H3A	0.2218	0.1146	−0.1213	0.031*	
H3B	0.2516	0.1145	0.0643	0.031*	
Cl1	0.091868 (19)	0.0000	0.5000	0.02019 (14)	

### Atomic displacement parameters (Å^2^)

**Table:**

	*U*^11^	*U*^22^	*U*^33^	*U*^12^	*U*^13^	*U*^23^
Zn1	0.01056 (16)	0.01421 (18)	0.01585 (18)	0.000	0.000	−0.00039 (15)
O1	0.0165 (7)	0.0220 (8)	0.0197 (8)	0.000	0.000	0.0020 (7)
N1	0.0158 (6)	0.0144 (6)	0.0188 (6)	0.0013 (5)	0.0016 (5)	−0.0005 (6)
C1	0.0132 (6)	0.0175 (8)	0.0197 (7)	0.0003 (6)	0.0009 (7)	0.0003 (7)
C2	0.0159 (7)	0.0209 (8)	0.0247 (8)	−0.0033 (6)	−0.0007 (6)	−0.0013 (7)
C3	0.0148 (7)	0.0274 (9)	0.0342 (9)	−0.0031 (6)	−0.0007 (7)	0.0014 (9)
Cl1	0.0188 (3)	0.0243 (3)	0.0174 (3)	0.000	0.000	−0.0004 (2)

### Geometric parameters (Å, °)

**Table:**

Zn1—N1^i^	2.1440 (13)	C1—C2	1.5227 (19)
Zn1—N1^ii^	2.1440 (13)	C1—C1^iii^	1.530 (3)
Zn1—N1^iii^	2.1440 (13)	C1—H1	1.0000
Zn1—N1	2.1441 (13)	C2—C3	1.526 (2)
Zn1—O1	2.2057 (16)	C2—H2A	0.9900
Zn1—O1^i^	2.2057 (16)	C2—H2B	0.9900
O1—H1O	0.831 (13)	C3—C3^iii^	1.522 (3)
N1—C1	1.4795 (18)	C3—H3A	0.9900
N1—H1A	0.871 (14)	C3—H3B	0.9900
N1—H1B	0.873 (13)		
			
N1^i^—Zn1—N1^ii^	80.65 (7)	Zn1—N1—H1B	108.4 (11)
N1^i^—Zn1—N1^iii^	99.35 (7)	H1A—N1—H1B	109.5 (16)
N1^ii^—Zn1—N1^iii^	180.00 (8)	N1—C1—C2	113.42 (12)
N1^i^—Zn1—N1	180.0	N1—C1—C1^iii^	108.68 (10)
N1^ii^—Zn1—N1	99.35 (7)	C2—C1—C1^iii^	110.85 (10)
N1^iii^—Zn1—N1	80.65 (7)	N1—C1—H1	107.9
N1^i^—Zn1—O1	89.79 (5)	C2—C1—H1	107.9
N1^ii^—Zn1—O1	90.21 (5)	C1^iii^—C1—H1	107.9
N1^iii^—Zn1—O1	89.79 (5)	C1—C2—C3	111.30 (13)
N1—Zn1—O1	90.21 (5)	C1—C2—H2A	109.4
N1^i^—Zn1—O1^i^	90.21 (5)	C3—C2—H2A	109.4
N1^ii^—Zn1—O1^i^	89.79 (5)	C1—C2—H2B	109.4
N1^iii^—Zn1—O1^i^	90.21 (5)	C3—C2—H2B	109.4
N1—Zn1—O1^i^	89.79 (5)	H2A—C2—H2B	108.0
O1—Zn1—O1^i^	180.00 (7)	C3^iii^—C3—C2	111.41 (11)
Zn1—O1—H1O	112.5 (13)	C3^iii^—C3—H3A	109.3
C1—N1—Zn1	109.77 (9)	C2—C3—H3A	109.3
C1—N1—H1A	108.5 (11)	C3^iii^—C3—H3B	109.3
Zn1—N1—H1A	112.7 (11)	C2—C3—H3B	109.3
C1—N1—H1B	107.9 (11)	H3A—C3—H3B	108.0

Symmetry codes: (i) −*x*, −*y*, −*z*; (ii) −*x*, *y*, *z*; (iii) *x*, −*y*, −*z*.

### Hydrogen-bond geometry (Å, °)

**Table:**

*D*—H···*A*	*D*—H	H···*A*	*D*···*A*	*D*—H···*A*
N1—H1A···Cl1^iv^	0.87 (1)	2.80 (1)	3.6139 (13)	156 (1)
N1—H1B···Cl1	0.87 (1)	2.70 (1)	3.5206 (14)	157 (1)
O1—H1O···Cl1	0.83 (1)	2.26 (1)	3.0857 (12)	173 (2)

Symmetry codes: (iv) *x*, *y*+1/2, −*z*+1/2.
